# A novel experimental approach for studying life-history traits of phytophagous arthropods utilizing an artificial culture medium

**DOI:** 10.1038/s41598-019-56801-4

**Published:** 2019-12-30

**Authors:** Kamila Karpicka-Ignatowska, Alicja Laska, Lechosław Kuczyński, Brian G. Rector, Mariusz Lewandowski, Ewa Puchalska, Anna Skoracka

**Affiliations:** 1 Population Ecology Lab, Institute of Environmental Biology, Faculty of Biology, Adam Mickiewicz University, Poznań, Poland; 2 USDA-ARS, Great Basin Rangelands Research Unit, Reno, NV USA; 3 Section of Applied Entomology, Department of Plant Protection, Institute of Horticultural Sciences, Warsaw University of Life Sciences – SGGW, Warsaw, Poland

**Keywords:** Model invertebrates, Ecology, Evolutionary developmental biology

## Abstract

Experimental approaches to studying life-history traits in minute herbivorous arthropods are hampered by the need to work with detached host plant material and the difficulty of maintaining that material in a suitable condition to support the animal throughout the duration of the test. In order to address this shortcoming, we developed a customizable agar-based medium modified from an established plant cell-culture medium to nourish detached leaves laid atop it while also preventing arthropods from escaping the experimental arena. The artificial culture medium was tested with two herbivorous mite species: the wheat curl mite (*Aceria tosichella*; Eriophyidae) and two-spotted spider mite (*Tetranychus urticae*; Tetranychidae). The proposed approach was a major improvement over a standard protocol for prolonged studies of individual eriophyid mites and also provided some benefits for experiments with spider mites. Moreover, the described method can be easily modified according to the requirements of host plant species and applied to a wide range of microherbivore species. Such applications include investigations of life-history traits and other ecological and evolutionary questions, e.g. mating or competitive behaviours or interspecific interactions, assessing invasiveness potential and predicting possible outbreaks. The approach presented here should have a significant impact on the advancement of evolutionary and ecological research on microscopic herbivores.

## Introduction

Organisms have evolved a great variety of life histories that are keys to understanding the action of natural selection and species diversity including complexities of their life cycles^1^. Life-history traits represent the timing and magnitude of investments in growth, reproduction and survival over an individual’s lifetime. However, resources that are available to any given organism are finite, thus, life history evolution is consistently constrained by trade-offs^1–3^. Since resource limitations and the resulting trade-offs operate at an individual level, life-history trade-offs should ideally be estimated at the level of the individual organism. However, due to logistical challenges, this is often not the case and life-history traits are assessed at the level of populations or cohorts^4–8^.

The ideal procedure for measuring life-history traits should involve keeping track of individual organisms and recording traits of interest throughout their lifetimes. To achieve this, specimens should be individually marked or spatially separated. However, for tiny organisms (e.g. various arthropods including mites) individual marking schemes are not feasible and thus the only option involves rearing individuals in separation. This poses its own technical challenges, especially for organisms like phytophagous arthropods that live in obligatory associations with their hosts, which are sources of food as well as shelter. Such host-associated interactions introduce an additional trophic level to be maintained during experiments, which presents a further logistical challenge. When tiny plant-feeding arthropods are reared on whole plants, they can hide within minute crevices in plant tissue or curled leaves, making their detection problematic. Ideally, they should be reared in arenas in which their presence, status, development, reproduction, and behaviour can be easily monitored.

Methods for maintaining and rearing individual arthropod specimens on plant fragments have been reported, e.g. maintenance of plant tissues on moist cotton balls or a hydrogel layer^9,10^, but such protocols often have drawbacks due to necrosis and other deterioration that occurs during incubation of the plant fragment. Therefore, such approaches are largely limited to short-term studies. Alternatively, plant fragments could be frequently replaced during an experiment although during frequent transfer of minute individuals, there is a risk of mortality or injury to the study subject. There are numerous possible modifications to the detached-leaf approach, e.g. where the leaf sample is placed directly on water or on a support other than cotton (e.g. poly-urethane^11,12^) but they do not prevent leaf necrosis, which is the largest drawback of this approach. Another method that has been used in experimental plant embryology and plant cloning is to put plant samples *in vitro* on a specific medium with agar and nutrients to prolong their lifespan^13–15^. This protocol has occasionally been applied to rearing phytophagous invertebrates^16–18^. However, the disadvantage of this method is that it requires antiseptic conditions that can only be assured by work in laminar flow cabinet to prevent contamination by fungi and bacteria and limiting the scale and accessibility of the experiment. In summary, no protocol currently exists to overcome the aforementioned limitations and allow for studies conducted on individual organisms with daily observations necessary for monitoring life-history traits or other important ecological characteristics such as mating or competitive behaviours and others intra- or interspecific interactions.

Tiny, plant-feeding mites represent a popular class of laboratory study systems for ecological and evolutionary experiments due to their environmental significance, e.g. as plant pests; their relatively short lifespans and generation time, which allows for rapid population growth and high densities; the possibility to obtain a desired number of generations, e.g. by manipulation of rearing temperature; and relatively low rearing costs^19–26^. Development of an effective method of rearing such organisms with the ability to track an individual’s fate across its entire lifespan would accelerate ecological and evolutionary research. Here we propose a method for maintaining and rearing tiny plant-feeding mites directly on plant fragments. Our method allows the plant tissue to remain viable for up to several weeks. At the same time, individual mites can be easily monitored, observed and manipulated. We tested the proposed methodology using the phytophagous mites *Aceria tosichella* (Keifer, 1969) (wheat curl mite; WCM) belonging to Eriophyidae and *Tetranychus urticae* (Koch, 1836) (two-spotted spider mite; TSSM) belonging to Tetranychidae, as study subjects.

This was accomplished through modifications of a standard Murashige and Skoog medium (MS medium)^27^ (a medium used for plant cell-culture; hereafter “artificial culture medium” or ACM), which allowed: (a) prevention of study subjects escaping the arena (via manipulation of agar concentration), (b) maintenance of healthy plant fragments (via addition of phytohormones) and, (c) prevention of fungal or bacterial contamination (via addition of antimicrobial agents). Next, we compared the effectiveness of this protocol with established methods (modified Munger cells, composed of a stack of Plexiglas plates for rearing eriophyid mites, and plant fragments put on wet cotton balls for tetranychid mites^28^) hereafter referred to as “standard”. We assessed: (a) the number of individual mites remaining in the plant arena after 24 hours; (b) the amount of time needed to set up an experiment; and (c) the overall cost (in terms of time and money) of the protocols. Finally, we demonstrated this method in an experiment assessing the developmental time and survival of WCM, including daily observations of individual mites.

## Methods

### Study system

Two minute phytophagous mite species, wheat curl mite *Aceria tosichella* (Eriophyidae) (WCM) and two-spotted spider mite *Tetranychus urticae* (Tetranychidae) (TSSM) were used as study subjects. Both species are globally distributed and are important crop pests; they both inhabit wild and cultivated plant species, causing serious damage to their hosts by inducing leaf chlorosis and organ malformations^29–31^. These species exhibit high potential for biological invasion and are expanding their ranges^29,32^. WCM and TSSM are popular model organisms for ecological and evolutionary studies including host-parasite interactions, genetic diversity, range expansion, host adaptation and dispersal and therefore they are commonly reared in many labs^22,30,32–37^.

WCM specimens used in these experiments were derived from a stock colony maintained for several years in the Population Ecology Lab, Faculty of Biology, Adam Mickiewicz University in Poznań, Poland. They were reared on bread wheat, *Triticum aestivum* L. var. “Muszelka” plants growing in pots from commercially available seeds. TSSM specimens were derived from a stock colony maintained for several years in the Section of Applied Entomology, Department of Plant Protection, Institute of Horticultural Sciences, Warsaw University of Life Science (SGGW). Spider mite specimens used for experimental purposes were reared on common bean, *Phaseolus vulgaris* L. var. “Ferrari” plants.

### New protocol for studying life-history traits of phytophagous mites

The two mite species were reared in arenas in which plant fragments (wheat and bean leaves) were placed on modified *in vitro* MS medium^27^. We applied substantial modifications that were designed to enhance the medium’s utility; e.g. to prolong leaf fragment viability and to reduce the risk of fungal and bacterial contamination. This artificial culture medium (ACM) was composed of basal ingredients used in MS medium: major (consisting of Macro A x100 and Macro B x100 solutions) and minor salts solutions (x200); and vitamin solution (x200)^27^, which are commercially available (East-Syntex, Poland). It was altered as follows. (i) Agar was added based on preliminary observations testing a range of concentrations from 1 g L^−1^ to 10 g L^−1^, at 1 g L^−1^ intervals. For each agar concentration we performed tests by transferring 10 mite individuals to a plant sample resting on the agar and then we counted their number after 24 and 48 hours. The desired agar concentration maintained leaf turgor and prevented mites from either sinking when they tried to leave the plant fragment or walking across the surface of the medium and escaping the arena. The standard concentration used in basal MS medium is 7 g L^−1^. For the experiments reported here, we used a concentration of 10 g L^−1^ for WCM and 2 g L^−1^ for TSSM. (ii) To promote cell division in the leaf fragments, the medium was supplemented with phytohormones as follows: 0.5 mL L^−1^ 1-naphthaleneacetic acid; 0.5 mL L^−1^ 6-benzylaminopurine; and 30 g L^−1^ sucrose. (iii) The addition of 10 mL L^−1^ IS10 preservative protected the medium from microbial contamination so the experiments could be conducted under non-sterile conditions, without using a laminar flow hood. The medium was prepared according to the following protocol (recipe for 100 mL of medium):

1. Add 2 mL of major salts (1 mL of Macro A and 1 mL of Macro B), 0.5 mL of minor salts and 0.5 mL of vitamin solutions to 70 mL of distilled water.
2. Add 0.05 mL of each phytohormone and 1 mL of preservative, whilst stirring.
3. Add the appropriate amount of agar (according to the studied species), plus 3 g of sucrose in the mixture and mix thoroughly.
4. Fill to 100 mL with distilled water.
5. Heat the medium in a microwave oven, stirring occasionally until it boils.
6. Cool the mixture down to 30–40 °C before pouring in order to avoid thermal shock to the plant.
7. Place the leaf fragment on the medium after cooling.

Artificial culture medium was poured into 6-well Plexiglas plates. Afterwards, plant fragments were placed on the medium: 5 × 5 mm wheat (*Triticum aestivum*) leaf fragments for WCM; and 10 × 10 mm bean (*Phaseolus vulgaris*) leaf fragments for TSSM. Size of plant fragments were selected according to the size of the mite species tested.

### Effectiveness of new approach in comparison with currently used methods

We compared the effectiveness of our new method of maintaining and rearing individual mites with two other methods commonly used for eriophyid mites^38^ and spider mites^20,39–41^.

For eriophyid mites, we used modified Munger cells adapted from Druciarek *et al*. (2014)^38^ composed of a stack of four 100 × 50 mm Plexiglas plates, in the following order: 2 mm thick bottom plate, a similar plate covered with tissue paper, a 5 × 100 mm wheat leaf fragment, 2 mm thick plate, with a 4 mm diameter hole in the centre sealed with plasticine, and 2 mm thick top plate, with a 10 mm ventilation hole, covered with fine muslin mesh. The stack was held together with rubber bands. In our case we used a Plexiglas top plate with a 4 mm diameter hole in the centre instead of a 10 mm diameter hole as proposed by Druciarek *et al*. (2014)^38^ as wheat leaves are narrower than the rose leaves used by the previous authors, thus the hole could be smaller to limit mite escape. Arenas for spider mites consisted of plant fragments put on wet cotton balls placed in Plexiglas plates^35^. Cotton between Plexiglas plates must be moistened every few days after daily inspection of their state.

There were two experimental groups for each mite species: one reared in arenas of the standard type and the second reared on ACM. Within each experimental group, we transferred 10 females of WCM (using an eyelash glued to dissecting needle) and 10 females of TSSM (using a small brush) from stock colonies to rearing arenas, each species in 10 replications, giving in total 100 females per species per experimental group. The experiment was conducted at room temperature (22–23 °C) and ~80% RH. Each arena represented an experimental unit.

**Figure 1:**
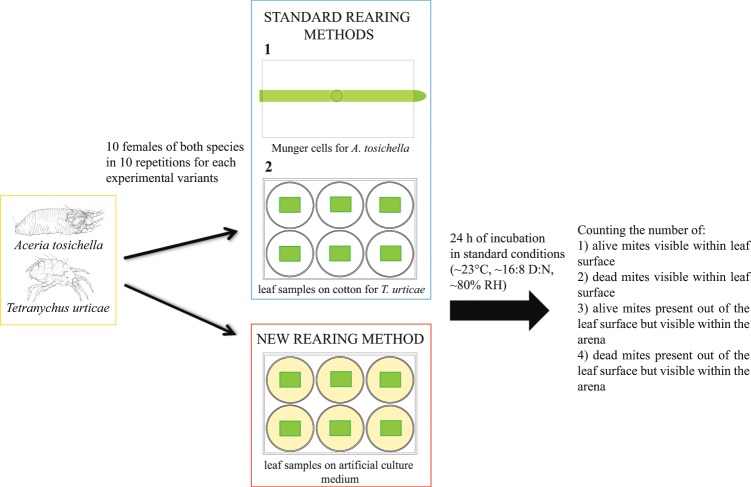
The experimental design for testing maintenance and survival rate of standard methods and ACM.

To compare the “standard” methods to ACM, we used two indices. The “maintenance effectiveness” index was calculated as the number of mites (either living or dead) found after 24 h in the arena divided by the total number of mites transferred (as described above). This index reflected the probability of retrieving data from a given individual; its complement was the proportion of mites that escaped the arena. The “survival rate” index was calculated as the number of living mites found after 24 h in the arena divided by the total number of mites transferred (Fig. 1).

To investigate whether there was an effect of rearing method and whether this effect is species-specific, a generalised linear model (GLM) was used with a quasi-binomial distribution for proportions and the logit-link function. The response variable was the proportion of individuals present after 24 h in each experimental trial and predictors were method (standard vs. ACM), species (WCM vs. TSSM) and their interaction. Effects were tested using type II likelihood-ratio chi-square tests^42^. We used pairwise post-hoc comparisons to determine significant differences between method-species combinations using estimated marginal means implemented in the emmeans R package^43^.

We estimated the overall cost and time needed to apply the compared methods. For this purpose the cost of all elements and ingredients needed to prepare each method for 10 experimental trials was calculated: the price of Plexiglas elements for standard methods (rearing in cages and on cotton); the price of 6-well Plexiglas plates and the price of ingredients needed to prepare ACM. To compare the overall time needed to set up the experiment for each method, we measured the time needed to: (i) prepare 10 arenas; (ii) transfer 10 females to each arena, and (iii) monitor mite presence on arena after 24 hours.

### Case study: developmental time and survival of wheat curl mite, WCM, assessed during daily observations

Individual females of WCM were transferred from the aforementioned stock colony to 5 × 5 mm wheat fragments placed on ACM, and incubated at 17 °C, 80 ± 5% RH and 16:8 (L:D) in growth chambers, in 28 replications. Females were monitored daily and removed from the arena after laying their first egg. The time of mite development from the first egg laid by an experimental female until the first egg of the next generation, and the time of individuals death was noted. To estimate developmental time the females that survived till the oviposition of first egg were included. Ninety-five-percent confidence intervals (95% CI) around mean egg-to-egg developmental time were calculated using bias-corrected and accelerated bootstrap^44^. Survival curves were estimated using the Kaplan-Meier method^45^. All analyses were done in R version 3.6^46^.

## Results

### Comparison of methods

**Figure 2:**
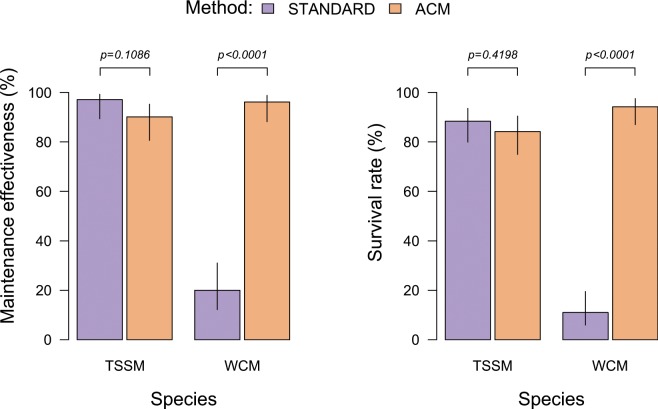
Comparison of maintenance effectiveness and survival rate between the “standard” and ACM methods for spider mites (TSSM) and eriophyid mites (WCM). Bars represent 95% confidence intervals around mean percentages.

There were significant effects for method, species, and their interaction on both maintenance effectiveness (ME) and survival rate (SR) (Table 1, Fig. 2). Both indices showed very similar and consistent patterns across treatments and species.

**Table 1:** Analysis of deviance table for a generalised linear model fitted to the proportion of all mites (dead or alive) present in the working arena (maintenance effectiveness) and the proportion of all living mites present in the working arena (survival rate) after 24 h of the experiment.

Effect	Maintenance effectiveness			Survival rate		
	Likelihood-ratio test	Df	p	Likelihood-ratio test	Df	p
Method	61.2	1	<0.0001	81.2	1	<0.0001
Species	64.3	1	<0.0001	61.4	1	<0.0001
Method × Species	40.0	1	<0.0001	62.9	1	<0.0001

When averaged across species, there was a clear difference between rearing methods (estimated marginal means for ME: z-ratio = 3.12, p = 0.0018, for SR: z-ratio = 6.28, p < 0.0001). The mean ME for the “standard” method was 59.1% (95% Confidence Intervals, CI: 45.4–71.9), whereas the mean ME for the ACM method was significantly higher: 93.1% (95% CI: 84.1–98.0). In case of SR, the mean for the “standard” method was 50.2% (95% CI: 36.6–63.8) and the mean for ACM was 89.2 (95% CI: 78.8–95.8).

When averaged across methods, both ME and SR differed significantly between the two mite species (ME: z = −3.68, p = 0.0002, SR: z = −4.18, p < 0.0001). For WCM, the mean ME was 58.6% (95% CI: 44.7–71.6), while for TSSM it was higher: 93.6% (95% CI: 84.6–98.3). A similar pattern was observed for SR: in case of WCM is was 58.6% (95% CI: 44.7–71.6) and 93.6% (95% CI: 84.6–98.3) in the case of TSSM.

However, there was a significant interaction between method and species for both considered indices (Table 1, Fig. 2). There was a substantial gain in ME when switching from the “standard” to the ACM method for WCM (z = 6.74, p < 0.0001); however, for TSSM the ACM method was not significantly different from the “standard” method (z = −1.60, p = 0.1086). For WCM, the ME of the “standard” method was 20.0% (95% CI: 11.8–30.4) and for the ACM it was almost five times higher: 96.1% (95% CI: 89.9–99.1). By contrast, for TSSM the ME of the “standard” method was 97.1% (95% CI: 91.4–99.5), and for the ACM method it was similar at 90.1% (95% CI: 81.7–95.6).

Exactly the same pattern was observed in SR: for TSSM it was similar when comparing the “standard” and ACM methods (z = −0.81, p = 0.4198), but significantly different for WCM, which performed better on ACM (z = 8.59, p < 0.0001). The mean SR for WCM kept on the “standard” method was 11.0% (95% CI: 5.6–18.7) compared to 94.2% (95% CI: 88.0–97.8) when using ACM. Survival of TSSM using the “standard” method was 88.3% (95% CI: 80.6–93.9) versus 84.2% (95% CI: 75.6–90.8) when using ACM.

Taking into account the cost associated with the need to purchase reagents, the up-front cost of the ACM method was high (see Supplementary Information 1). However, a minimal amount of reagents allowed for the preparation of many ACM arenas (around 100), so the cost of 10 arenas was relatively low (about 4.5 EUR). The most expensive method was the use of the current standard rearing arenas (modified Munger cells)^38^, while the costs of preparing standard spider mite arenas with cotton and ACM were similar. The preparation of 10 ACM arenas was less time-consuming for WCM (22 minutes) compared to the “standard” method (31 minutes), but for TSSM, the time spent on the “standard” method was only six minutes. The time needed to transfer 10 specimens of both eriophyid and spider mites to the ACM arena was ca. 2 minutes (for WCM mean: 2 min., range: 2–2 min., n = 10; for TSSM mean: 2.1 min., range: 2–3 min., n = 10), similar to that for transferring spider mites to the cotton arena (mean: 2 min., range: 2–2 min., n = 10). Transferring eriophyid mites to the rearing arena in Munger cells took on average 5 min and 18 seconds (mean: 5.3 min., range: 3–7 min., n = 10). This almost 3-fold difference in time when transferring eriophyid mites to the rearing cages compared to *in vitro* arenas emerged from the difficulty of eyelash manipulation and putting the mite specimen in the 4 mm diameter hole. There was no difference between “standard” and ACM methods in the time needed to check the arena after 24 hours; for all tested methods it was 1 minute.

### Developmental time and survival of wheat curl mite (WCM) assessed during daily observations

**Figure 3:**
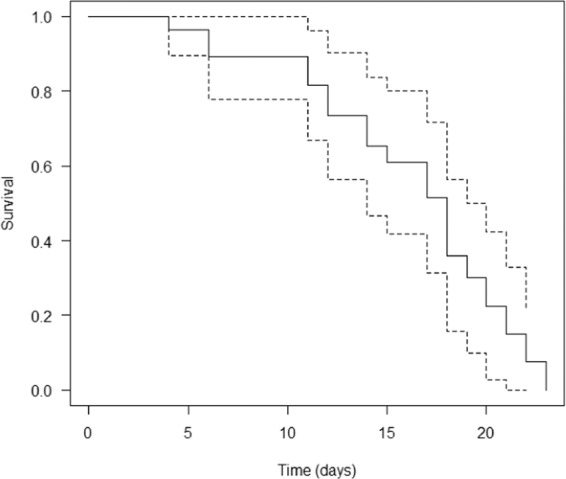
Kaplan–Meier survival curve for WCM at 17 °C. Dashed lines represent 95% confidence band.

The ACM protocol allowed us to simply and easily conduct daily observations of individual mites without destroying the host plant. Egg-to-egg development of WCM at 17 °C lasted on average 19.0 days (95% CI: 17.6–20.0, n = 5), and the median survival time was 17 days (CI: 14–19, n = 28). Figure 3 depicts a Kaplan-Meier survival curve for WCM when reared at 17 °C. Until the 4^th^ day of observation, the survival rate was 100% (Fig. 3) and the maximum life span was 23 days.

## Discussion

Ecologists increasingly use experimental methods in order to explain the mystery of the remarkable diversity of life. One way to obtain broader knowledge about the biology and ecology of a given species is to study its life-history traits (e.g. fecundity, survival, lifespan, etc.) or behaviour (e.g. mating, inference competition, etc.). The case is even more crucial as it relates to species of high economic importance, for example phytophagous pests, as proper estimation of life-history traits may help in assessing their invasive potential and predicting their possible outbreaks. Such studies are of current interest and have been recently conducted on different phytophagous taxa^7,47,48^. However, experimental investigations on minute arthropods can be problematic due to their small size, especially if the research approach requires direct observation of single individuals. Studying life-history traits in herbivorous organisms is even more difficult as it requires the inclusion of an additional trophic level, the host plant. It is problematic to maintain detached leaves if the medium is not suitable or lacks optimal mineral nutrients, in which case plants may show stunted and abnormal growth or tissue degradation such as chlorosis^49^. However, if the culture medium is composed correctly, the time between plant passages may increase to as many as 30 days, depending on environmental factors (e.g. light type or humidity)^50-53^.

In our study we introduce a novel rearing method with leaf fragments maintained on an artificial culture medium (ACM) in order to efficiently rear and monitor individual microherbivores. We demonstrated its maintenance effectiveness and survival rate using representatives of two major taxa of phytophagous mites: the eriophyid mite *Aceria tosichella* and the spider mite *Tetranychus urticae*.

The ACM provides basic elements for plant growth as well as phytohormones (auxin and cytokinin) that stimulate plant cells to divide, maintaining functional plant tissues similar to those of a living plant^54,55^. It has been shown that exogenous application of auxins (e.g. 1-naphthaleneacetic acid: NAA) can significantly delay chlorophyll loss and protein degradation in detached leaves of various plant species^56^, whereas application of cytokinin 6-benzyladenine may inhibit senescence of cut leaves^57^ and it plays important role in tissue rejuvenation^58,59^. According to Abro *et al*. (2004)^60^ application of NAA has no significant influence on cotton infestation by leafhoppers, thrips, whiteflies, or lepidopteran larvae. However, the harmful effect of NAA on fecundity and the intrinsic rate of natural increase of aphids feeding on wheat was observed after the application of this phytohormone at a concentration of 150 mg/L^−1^. Lower concentrations (50 and 100 mg/L^−1^) had no effect on development as well as reproductive parameters of these aphids^61^. Unfortunately, the potential harmful effect of benzyladenine (BAP), as well as plant preservatives mixture (IS10), was not studied. However, the low concentrations of these substances in ACM and the lack of direct contact of the reared organisms with the medium seems to exclude its potential harmful effect. This can be supported by the fact that media containing these substances are commonly used to study plant-invertebrate interactions^62–64^. The composition of the medium, especially types and concentrations of hormones and agar, may be easily changed based on requirements of the plant species under study^65–70^. Thus, different plant samples can be maintained for a long time without excessive intervention, thereby reducing herbivore stress and mortality caused by frequent transfers between experimental arenas and improving the quality of the obtained data. This protocol increases the reliability of the results while allowing observations of individual specimens. In addition, arenas with ACM do not require use of laminar flow chambers and thus the method can be applied to various ecological and evolutionary studies without sterile conditions.

The method we propose is based on cut plant material; with the proper composition, the medium preserves the plant fragment for an extended period of time and allows investigation of a variety of microherbivore traits and behaviours. The use of cut plant fragments is essential for monitoring development, reproduction or behaviour of tiny organisms, invisible to naked eye, especially those that are refuge-seeking and hide within folds, crevices, or leaf whorls whenever possible. Such long-term observations of individuals or small colonies of microherbivores would simply not be possible on whole plants. However, one limitation of the ACM method is that the use of cut plant fragments cannot account for a whole plant’s organismal response to the microherbivore’s feeding. This would be particularly important in studies in which both plant and herbivore data are recorded, rather than only the herbivore’s behaviour or life-history traits. Measurements of changes to a cut plant fragment in response to microherbivore feeding could address part of this concern but we have not explored this avenue to date.

Due to their economic importance, eriophyid mites are subjects of various ecological studies investigating mite characteristics that can be useful in predicting their distribution and outbreaks but these are often conducted using cohorts of specimens infesting whole plants^21,23,71^. However, due to their minute size, eriophyids can be difficult to detect and monitor when reared on whole plants. We demonstrated here that ACM is significantly more effective and practical in experimental studies conducted on eriophyid mites compared to a standard method based on modified Munger cells (Fig. 2, Table 1) because as the leaf surface is tightly fixed to the moist *in vitro* medium, specimens cannot get under the leaf surface; if they walk off the leaf fragment they may be easily retrieved from the medium and put back on the leaf. By contrast, although many efforts have been undertaken to assess life-history traits of eriophyoid mites in standard Munger cells (with daily data collections), these efforts have largely failed because mites escaped from the arenas in the cages. Moreover, reduced hole diameter in the cage results in difficulties in transferring mites, as well as their detection during experimental treatments. As such, there are no available life-history data for WCM, such as developmental time and survival, to compare with data presented here utilizing ACM.

For eriophyid mites, ACM was also less expensive and time-consuming to prepare than the standard protocol. Comparing the cost of ingredients needed to prepare rearing arenas with ACM we showed that ACM is less than half as expensive as Munger cells (see Supplementary Information 1). However, it should be taken into account that those cages can be used repeatedly, which reduces the long-term costs of this method.

While very advantageous for maintaining phytophagous eriophyid mites, compared to the standard method, ACM appeared to be similarly effective to the standard method when applied to spider mites (Fig. 2). However, it may be more advantageous in comparison to the standard method for conducting experiments with spider mites, during which observations must be made over the course of an individual’s lifespan, including the following: longevity; number, size, and sex ratio of offspring; age- and size-specific reproductive investments; and survival tables. It should be easier to examine those traits using ACM since, with the proper agar concentration, mites would not drown in the medium, which is a common cause of death when using moistened cotton balls. As such, the concentration should be adjusted so the agar surface is firm enough to keep study subject from drowning in the medium, while at the same time soft enough to prevent them from crawling across it. This advantage, in our opinion, may improve the experiment’s effectiveness and allow for using a reasonable number of individuals needed to accomplish the study (with a reduced need for replacing dead specimens). However, the ACM entails a higher cost in comparison to the standard approach (see Supplementary Information 1) and is more time-consuming than rearing spider mites on cotton balls.

Taking into consideration both the benefits and drawbacks of ACM relative to standard approaches, the artificial culture medium method has many clear advantages, chief among them being simplicity, flexibility, and stability. It may be applied to a wide range of ecological and evolutionary investigations that require direct observation of phytophagous arthropod individuals. We have shown here that ACM is particularly useful for studies of eriophyid mites. It can be used in assessing life-history traits and is also ideal for intra- and inter-specific behavioural observations, for example interference competition or mate choice. All of these investigations could be conducted under different conditions by manipulating biotic (e.g. host plant species, the presence of competitors or conspecifics) and abiotic (e.g. temperature, photoperiod) factors.

## Supplementary information

Supplementary information.

## Data Availability

The datasets generated and analysed during the current study are available in the Zenodo repository under: 10.5281/zenodo.3415949.
