# Supplementary material for: A novel experimental approach for studying life-history traits of phytophagous arthropods utilizing an artificial culture medium
Source: Sci Rep. 2019 Dec 30;9:20327. doi: 10.1038/s41598-019-56801-4 (PMC6937311; doi:10.1038/s41598-019-56801-4)
Supplement: Supplementary file 1 — Supplementary information. [file 41598_2019_56801_MOESM1_ESM.pdf]

Supplementary Information 1

A novel experimental approach for studying life-history traits of phytophagous arthropods utilizing an artificial culture medium

Kamila Karpicka-Ignatowska[1], Alicja Laska[1*], Lechosław Kuczyński[1], Brian G. Rector[2], Mariusz Lewandowski[3], Ewa Puchalska[3], Anna Skoracka[1]

[1]Population Ecology Lab, Institute of Environmental Biology, Faculty of Biology, Adam Mickiewicz University, Poznań, Poland
[2]USDA-ARS, Great Basin Rangelands Research Unit, Reno, NV, USA
[3]Section of Applied Entomology, Department of Plant Protection, Institute of Horticultural Sciences, Warsaw University of Life Sciences – SGGW, Warsaw, Poland

Supplementary Information 1 The comparison of costs needed to prepare each method for rearing phytophagous mites

A. Cost breakdown for preparing 10 modified Munger cells for WCM rearing

**Element**	**Cost of one element [EUR]**	**Number of elements needed**	**Cost for preparing 10 trials [EUR]**
Plexiglas plates without a hole	0.19	20	3.8
Plexiglas plates with a hole	0.23	20	4.6
Tissue paper	0.01	10	0.1
Muslin mesh 10μm	0.19	10	1.9
Plasticine	0.002	10	0.02
Rubber bands	0.002	20	0.04
**Total cost**			**10.46**

B. Cost breakdown for preparing 10 TSSM arenas with cotton on water

**Element**	**Cost of one element [EUR]**	**Number of elements needed**	**Cost for preparing 10 trials [EUR]**
6-well Plexiglas plates	1.74	2	3.48
Cotton	0.01	10	0.1
**Total cost**			**3.58**

C. Cost breakdown for preparing 10 arenas filled with artificial culture medium (comparison made for 10g L^{-1} agar concentration)

**Element or ingredient**	**Cost of one element/ ingredient [EUR]**	**Number of elements or concentration needed**	**Cost for preparing 10 trials (100 mL of medium) [EUR]**
6-well Plexiglas plates	1.74	2	3.48
Basal MS medium ingredients	18.58 (400 mL)	3 mL	0.19
Agar	9.29 (100 g)	1 g	0.09
1-naphthaleneacetic acid	3.48 (10 mL)	0.05 mL	0.02
6-benzylaminopurine	3.48 (10 mL)	0.05 mL	0.02
IS10 preservative	46.43 (100 mL)	1 mL	0.46
Sucrose	0.69 (1000 g)	3 g	0.02
Distilled water	2.32 (5000 mL)	100 mL	0.05
**Total cost**			**4.33**

All costs were calculated based on the prices of materials in Poland, where these experiments were conducted. Prices may vary in other countries but we assume that between-country price fluctuation will be similar for all components of the tested protocols and thus not affect the comparison made here.
